# Vaccines, trust and European public health

**DOI:** 10.2807/1560-7917.ES.2018.23.17.18-00210

**Published:** 2018-04-26

**Authors:** Andrea Ammon, Xavier Prats Monné

**Affiliations:** 1 European Centre for Disease Prevention and Control (ECDC), Stockholm, Sweden; 2 Directorate-General for Health and Food Safety (DG SANTE), European Commission, Brussels, Belgium

**Keywords:** vaccines, vaccine-preventable diseases, public health

Vaccines have made a major contribution to the prevention and control of communicable diseases. They are considered as one of the successes in medicine and a cornerstone of public health. They have saved, and continue to save, human lives and prevent debilitating and deadly diseases, reduce illness and have improved the health and life expectancy of populations. Vaccination has led to the eradication of smallpox, and is bringing us closer than ever to the global elimination of poliomyelitis, measles and rubella. It prevents countless deaths from many other diseases such as pertussis, diphtheria, invasive *Haemophilus influenzae* b and meningococcal infections.

Despite these achievements, national vaccination programmes in Europe are facing numerous challenges. Sizeable population groups across the European Union (EU) are hesitant towards or even refuse vaccination, which has led to a decline in vaccination coverage for several vaccines and continued outbreaks of vaccine-preventable diseases such as measles, with cases spreading from one country to others, causing avoidable deaths [1,2]. This situation is of great concern and continuous attention, efforts and engagement are required by all those concerned: citizens, medical and public health professionals and politicians.

On 13 September 2017, Jean-Claude Juncker, President of the European Commission, reflected in his annual *State of the European Union* address, on suboptimal vaccination coverage, stating that *‘It is unacceptable that in 2017 there are still children dying of diseases that should long have been eradicated in Europe* […] *No ifs, no buts* [...] *Avoidable deaths must not occur in Europe’* [3].

However, these deaths still occur, most often in unvaccinated individuals or people that could have been protected by sufficient population immunity. In 2017, up to 80% of adolescents and adults who contracted measles had not been vaccinated, although measles vaccination is available for free in all EU countries [4]. In recent and ongoing measles outbreaks, transmission in healthcare settings has played an important role, with many of those affected being healthcare workers who should have been vaccinated to protect themselves and their patients from disease [2]. Moreover, healthcare workers are important ambassadors of vaccination.

## Erosion of trust in vaccines

Public trust in vaccines is a crucial issue, and any erosion of this trust leads to lower uptake and has negative consequences for vaccination programmes. A multitude of factors undermine public trust in vaccines and affect vaccination uptake. These include lack of information, lack of knowledge, not accessing trusted and credible information sources, questioning of the actual need for specific vaccines and concerns about vaccine safety fuelled by public debates.

As illustrated in this issue of *Eurosurveillance* in an article by Rey et al., safety concerns in particular can have serious effects on the willingness of parents to vaccinate their children and of people to get vaccinated themselves [5]. What would Louis Pasteur think, should he be reading the outcome of the survey by Rey et al? What has led to such strong concerns and made vaccination unpopular and questioned to such an extent? History has shown that it takes only one piece of widely communicated bad research to put a global elimination effort at stake. In this issue, an interview with Stanley Plotkin, one of the pioneers of vaccination, reflects on this and other points [6].

Fear and concerns related to specific vaccines and population groups may not hold true for others. It is therefore important to understand the drivers behind the hesitancy for each of them and tailor interventions adequately.

## Establishing public confidence

Monitoring the safety of a vaccine is of primary importance for public health authorities. In this issue, Mentzer et al. present post-marketing surveillance of adverse event reports of the recently introduced serogroup B meningococcal (MenB) vaccination [7]. They were able to demonstrate that there were no signals contesting the safety of this vaccine. That systematic monitoring of safety is a continuous and legal requirement for all vaccines, needs to be brought to the attention of EU citizens with the potential to re-instigate trust.

Public health policies, including vaccination programmes, are under the responsibility of the individual EU Member States, as are approaches to ensure adherence of citizens to those policies. In their article, Lévy-Bruhl et al. share the background and expected outcome of extending the number of vaccines that are mandatory in France [8]. The change in the law followed a period of public consultation and citizen engagement and was deemed best adapted to the situation in the country. It is one example of engaging citizens in order to address questions and concerns and find solutions that have a solid basis.

## Joining efforts to restore trust in the life-saving potential of vaccinations

Given that vaccine-preventable infectious diseases are not confined within national borders, and that one country’s immunisation weakness puts at risk the health and security of citizens across the EU, the added value of acting together at EU level is indisputable.

On 26 April 2018, following a period of public consultation and discussions with stakeholders, the European Commission presented a proposal of a Council Recommendation for strengthened cooperation against vaccine-preventable diseases, together with a Communication to the European Parliament, the Council and the European Economic and Social Committee and the Committee of the Regions. This proposal is an ambitious call for cooperation and specific actions with the aim to increase vaccination coverage and to reduce inequalities and gaps in immunisation by ensuring that everyone in the EU has access to vaccination. (Box).

Box **Selected actions from the proposed Council Recommendations for strengthened EU cooperation in the area of vaccine hesitancy**

Develop and implement national and/or regional vaccination plans by 2020, including a target of at least 95% vaccination coverage for measles;
Introduce routine checks of vaccination status and regular opportunities to vaccinate across different stages of life, for example in schools and workplaces;
Establish a European vaccine information sharing system (EVIS) to gather knowledge to develop guidelines for a core EU vaccination schedule by 2020;
Present options for a common vaccination card that can be shared electronically across borders;
Establish an online European vaccination information portal by 2019 to provide objective, transparent and updated evidence on the benefits and safety of vaccines;
Equip all healthcare workers with the necessary tools and training to confidently deliver vaccinations and address hesitant behaviour;
Convene a coalition for vaccination to bring together European associations of healthcare workers as well as relevant students’ associations in the field to commit to delivering accurate information to the public, combating myths and exchanging best practice;
Mitigate risks of shortages by developing a virtual EU data warehouse on vaccine needs to facilitate voluntary exchange of information on available supplies and shortages of essential vaccines; and
Strengthen partnerships and collaboration on vaccination with international partners, considering that infectious diseases do not respect national borders and that vaccination is a global health priority.

EU: European Union.For more information see [9].

In addition to the proposed Council Recommendations, a Joint Action on vaccination with 24 participating countries will introduce a number of practical measures to improve national vaccination uptake [9]. This dual approach, which combines actions at both the national and the EU level, should lead to marked improvements in the overall vaccination coverage and to better control of vaccine-preventable diseases in Europe.

The European Immunisation Week 2018 provides a good opportunity for communicating these important initiatives to the public health community.
